# An after hours gp clinic in regional Australia: appropriateness of presentations and impact on local emergency department presentations

**DOI:** 10.1186/s12875-017-0657-6

**Published:** 2017-09-11

**Authors:** Kristy Payne, Tegan Dutton, Kate Weal, Maree Earle, Ross Wilson, Jannine Bailey

**Affiliations:** 1 Bathurst Rural Clinical School, School of Medicine, Western Sydney University, Bathurst, NSW Australia; 2 Marathon Health, Bathurst, NSW Australia

## Abstract

**Background:**

After hours general practice clinics provide medical attention for clients with non-emergency situations but are seeking immediate treatment and unable to wait for a general practitioner during routine opening hours. Evidence on the impact that after hours clinics have on emergency department presentations is equivocal. This study explored outcomes of the Bathurst After Hours General Practice Clinic (BAHGPC). Specifically it examined: clients’ perceived urgency of, and satisfaction with their presentation to the BAHGPC; general practitioners’ perception of the appropriateness of presentations to the BAHGPC; and whether the frequency of non-urgent and semi-urgent emergency department presentations at Bathurst Base Hospital has changed since the opening of the BAHGPC.

**Methods:**

Clients presenting to the BAHGPC from 01/02/2015 to 30/06/2015 were asked to participate in the client presentation survey and follow-up satisfaction survey. General practitioner surveys were completed for individual clients from 01/12/2014 to 30/06/2015 to document the appropriateness of each presentation. Descriptive statistics are used to describe survey responses. Thematic analysis was applied for qualitative responses. Emergency department presentations were retrieved from the Emergency Department Data Collection. A comparison of presentations in the two years prior and subsequent to the opening of the BAHGPC was conducted using independent T-tests and Chi-square tests to compare mean presentations and proportional data for the different time periods examined.

**Results:**

Most clients (76%) presenting to the BAHGPC classified their visit as essential. General practitioners considered most presentations to be appropriate (87%). Sixty percent (60%) of clients would have gone to the emergency department had the BAHGPC not been operational. Client satisfaction was high and 99% would use the clinic again. A significant reduction in total non-urgent presentations to the Emergency Department occurred in the two years since the opening of the BAHGPC clinic compared to the two years prior (418.5 vs. 245.5; *P* < 0.05).

**Conclusions:**

There was concordance between general practitioners and clients regarding the appropriateness of presentations to the BAHGPC. The findings of this study highlight that after hours general practitioner clinics are an essential service in regional areas and contribute to reducing the burden of non-urgent presentations to the local emergency department.

**Electronic supplementary material:**

The online version of this article (10.1186/s12875-017-0657-6) contains supplementary material, which is available to authorized users.

## Background

Acute care services, both in Australia and internationally, are coming under increasing pressure. This is partly due to an increased burden of chronic disease, an ageing population and an overall reduction in general practitioner working hours, particularly in the after hours period [1–3]. In recent years in Australia, the average annual increase in the emergency department (ED) presentation rate [4] has been consistently higher than that of population growth [5]. In addition, it has been estimated that some 10 to 40% of ED presentations represent general practice clients more suited to primary care than acute care [6–11].

This increasing ED workload, and concerns about the appropriateness of some ED presentations, has prompted the implementation of Government initiatives to increase the availability and accessibility of after hours primary care. A recent systematic review [3] identified six models of after hours primary care including minor injury units, walk in centres, telephone triage and advice centres, ambulance officer managed care, the integration of a general practitioner within the ED team, and General Practice (GP) cooperatives. GP cooperatives, otherwise known as after hours GP clinics, provide medical attention for people who are seeking immediate treatment and cannot wait to see their regular general practitioner during their routine opening hours. They are designed to cater for people who do not have an emergency situation, but who would otherwise be likely to access their local ED for treatment due to the perceived urgency of their complaint [2]. International and Australian evidence on the impact of after hours GP clinics on low acuity emergency department presentations is equivocal [3].

One aspect of after hours GP clinics that has received little attention is the appropriateness of presentations to these clinics, both from the client and the general practitioner perspective. Only one study, conducted in the Netherlands, has addressed this and found that up to two-thirds of non-urgent presentations to GP cooperatives were “medically unnecessary” and could have been managed by their regular general practitioner during routine working hours, or could have been managed by the client themselves without professional care [12]. Two key limitations of this study were that the client presentation survey was conducted in the post-consultation period, hence relying on client recall and also leading to low response rates, and the appropriateness of their presentation was judged by two general practitioners retrospectively using only the client survey data. Further examination of after hours GP clinic presentations is therefore warranted to determine whether the presence of after hours GP clinics is providing a suitable alternative to the ED for low acuity clients.

The primary aims of this study, therefore, were to examine (a) the clients’ perceived urgency of their presentation to the after hours GP clinic, (b) the general practitioners’ perception of the appropriateness of each presentation to the after hours GP clinic, and (c) client satisfaction with the service. A secondary aim was to examine the frequency of semi-urgent and non-urgent presentations to the local ED, and compare data from the two years prior to the opening of the after hours GP clinic, to the two years since its opening.

## Methods

### Setting

The Bathurst After Hours General Practice Clinic (BAHGPC) is located next door to the Bathurst Base Hospital in the regional town of Bathurst, New South Wales, Australia. It is a bulk-billing, walk-in clinic; meaning that the Australian Government’s Medicare benefit covers full payment of the service and there are no out of pocket expenses for clients and no prior appointments can be made. Opening hours are 3 pm to 7 pm on weekends and public holidays (excluding Christmas Day and Good Friday). The clinic is routinely staffed by a receptionist, registered nurse and a general practitioner. The BAHGPC has been in operation since the 1st December 2012 and is the only after hours service in the local area.

### Study design

A survey study was conducted using a volunteer sample of participants. All clients who presented to the BAHGPC during the period 1st February 2015 to 30th June 2015 were eligible to participate in the client presentation survey. For presentations where the client was aged 18 years or older, the client was invited to participate. For presentations where the client was under 18 years of age, the client’s accompanying parent or guardian was invited to participate and complete the survey on behalf of the client, provided they were themselves aged 18 years or older. Clients were also asked to provide their contact details if they consented to being contacted over the phone for a follow-up satisfaction survey. This information was recorded separately so as to maintain the anonymity of their survey responses. The general practitioner appropriateness of presentation survey was conducted during the period 1st December 2014 to 30th June 2015. All general practitioners at the clinic were encouraged to complete this survey for all client encounters in the study period.

### Client presentation survey

The four item, anonymous client survey was administered by the clinic nurse and was completed by all consenting participants prior to their consultation. The survey asked for the clients’ reason/s for presenting to the clinic, where they would have sought treatment if the clinic was unavailable at that time, willingness to pay for the clinic, and their health insurance status (Additional file 1).

### Client follow-up satisfaction survey

The client follow-up satisfaction survey was conducted over the phone by the BAHGPC administration staff within two weeks following the client’s clinic presentation. Verbal consent was obtained from all participants before the survey was administered. Clients were asked to rate how satisfied they were with the outcome of their visit on a scale of 0 (very dissatisfied) to 10 (very satisfied), whether their issue was resolved, whether they would use the clinic again, whether they would recommend the clinic to others, and any additional comments (Additional file 2).

### General practitioner appropriateness of presentation survey

The three item general practitioner appropriateness of presentation survey was completed immediately following a client consultation (Additional file 3). General practitioners were asked to classify the appropriateness of the presentation and to rate the appropriateness of the presentation on a scale of 0 (not appropriate) to 5 (absolutely essential). They were also asked to identify the medical outcome identified, which the researchers categorised into one of ten presentation categories, namely: ear, nose and throat; respiratory; genito-urinary; gastrointestinal; neurological; cardiovascular; dermatological; psychological; documentation; and other.

### Examination of emergency department presentations

Semi-urgent and non-urgent ED presentations (Australasian triage categories 4 and 5, respectively) at Bathurst Base Hospital were retrieved from the New South Wales Emergency Department Data Collection. Data was extracted for the two years prior to the opening of the BAHGPC (1st December 2010 to 30th November 2012) and the two years subsequent to the opening of the BAHGPC (1st December 2012 to 30th November 2014). Only those presentations occurring between the hours of 12:00 pm Saturday through to 8:00 am Monday were examined. This time frame was chosen as it is during this period that there are no other GP clinics open in the local area (i.e. some GP clinics are open Saturday morning up until 12 pm and reopen on Monday at 8 am) and the only options for clients are the BAHGPC or the local ED. Non-identifiable data retrieved included time of presentation, triage category, age, and sex.

### Statistical analysis

Descriptive statistics were used to describe quantitative survey responses and ED presentation data. Independent T-tests were used to compare mean ED presentations during the two time periods examined. Chi-square tests were used to compare proportional data for the different time periods examined. Statistical analyses were conducted using IBM SPSS Statistics (Version 22.0). Statistical significance was set at *P* < 0.05. The qualitative responses on the client follow-up survey were reviewed by two researchers to familiarise themselves with the data. The key themes of positive, negative and neutral service comments were identified and used to categorise the responses.

## Results

### Presentations to the Bathurst after hours gp clinic

In the first two years of operation (1st December 2012 to 30th November 2014) there were 2125 presentations to the BAHGPC. Of these, 1205 (57%) were female and 920 (43%) were male. During the five month client study period (1st February 2015 to 30th June 2015), there were 827 clinic presentations, of which 62% were female and 38% were male. A total of 219 clients who presented to the clinic during this time frame participated in the client presentation survey. However, 14 were incomplete and not included in the analysis, resulting in a total of 205 surveys; a 25% response rate. The follow-up satisfaction survey was completed by 219 clients, a 26% response rate. During the period 1st December 2014 to 30th June 2015, 452 general practitioner surveys were completed by treating doctors, a response rate of 55%.

Three-quarters of all clients surveyed (76%; 155) deemed their visit to the BAHGPC as essential (Table 1). Sixty per cent (60%; 125) indicated that they would have presented to the ED had the BAHGPC not been operating at the time of their visit, of which 86% (107) classified their visit as essential. A quarter of respondents (27%; 55) indicated they would have waited until the following week to see their own general practitioner rather than present to the ED (Table 1), however 58% (32) of these respondents still deemed their visit as essential. Twenty-eight per cent (28%; 49) of participating clients had a health care card and 44% (75) had private health insurance (Table 1). One-quarter of respondents (25%; 52) reported that they would not use the BAHGPC if they had to pay for the service, i.e. if it was not bulk-billed. Of the clients who said no, only two gave a reason, being: *“Depends on acuity of presentation”*; and *“just in case I didn’t have the money at the time”*. Of the 75% that would be willing to pay for the service, 56% (87) would be willing to pay the same amount they currently pay their general practitioner, and 39% (60) would be willing to pay a small co-payment (less than $10). There was no significant association between possession of a health care card and willingness to attend the BAHGPC if the client had to pay for the service, i.e., it was not bulk-billed (X^2^ 1.344, *P* = 0.246). However, those with private health insurance were significantly more likely to indicate a willingness to attend the BAHGPC if they had to pay for the service (i.e. if it was not bulk-billed), compared to those who did not have private health insurance (X^2^ 11.926, *P* < 0.001).

**Table 1:** Client perceptions when presenting at the Bathurst After Hours General Practice Clinic

	Number	Percentage (%)
Main reason for attending the after hours clinic: (*n* = 205)		
Essential visit (had to come)	155	76%
Convenient	18	9%
Couldn’t get an appointment with a regular GP	16	8%
Only to get a script (out of my medication)	8	4%
Requested by the hospital ED to attend	1	0%
Requested by the GP to attend	0	0%
Other	7	3%
If the after hours clinic was not operational, the client would: (n = 205)^a^		
Go to the Hospital ED	125	60%
Wait until next week to see the client’s own GP	55	27%
Try and consult another GP in town	22	11%
Visit a pharmacist and try to get medication	3	1%
Other	3	1%
If the client had to pay for the clinic, would they use the service: (n = 205)^b^		
Yes:	154	75%
If yes, what is the maximum the client would be willing to pay: (*n* = 154)		
The same amount you would pay at your GP	87	56%
A small co-payment (less than $10)	60	39%
More than you would pay at your GP	4	3%
No response/missing data	3	2%
No	52	25%
Does the client have a health care card: (*n* = 175)		
Yes	49	28%
No	126	72%
Does the client have private health insurance: (*n* = 170)		
Yes	75	44%
No	95	56%

^a^More than one response provided by 3 clients; all responses are included in the analysis

^b^One respondent answered both yes and no; all responses are included in the analysis. Abbreviations: General Practitioner (GP), Emergency Department (ED)

From the perspective of the general practitioner, the majority of presentations to the BAHGPC were deemed appropriate (85%; 383) and an additional 2% (7) were considered appropriate *and* needed a referral to the local hospital or ED (Table 2). When queried about how essential the presentations to the BAHGPC were on a scale of 0 to 5 (0 being not appropriate and 5 being absolutely essential), 28% of presentations to the BAHGPC were rated by general practitioners as 5 (absolutely essential), 41% as 4, and 17% as 3 (Table 2). A total of 14% of presentations were rated on the lower end of the scale (0, 1 or 2). The most common presentations recorded by general practitioners were for an ear, nose and throat problem (20%), a dermatological problem (17%) or a respiratory problem (17%) (Table 2).

**Table 2:** General practitioner perceptions of presentations at the Bathurst After Hours General Practice Clinic

	Number	Percentage (%)
Classification of presentations to the Bathurst After Hours GP Clinic: (*n* = 449)		
Consultation was appropriate	383^a^	85%
Consultation was appropriate *and* needed a referral to hospital/ED	7	2%
Consultation was appropriate *and* was requested by clients GP during the week	0	0%
Consultation only for a script	9	2%
Consultation for an administrative reason	10	2%
Consultation for drug-seeker	3	1%
Consultation because it was convenient for the client	29^b^	7%
Other	13	4%
GP perception of appropriateness of presentation: (*n* = 452)		
5 (Absolutely essential)	125	28%
4	186	41%
3	77	17%
2	33	7%
1	17	4%
0 (Not appropriate)	14	3%
Primary reason for presentation: (n = 452)		
Ear, nose and throat	91	20%
Dermatological	77	17%
Respiratory	76	17%
Gastrointestinal	39	9%
Genito-urinary	34	8%
Documentation	20	4%
Neurological	9	2%
Psychological	8	2%
Cardiovascular	3	1%
Other – including dental, injury, immunisation	95	21%

*Abbreviations: GP* General Practitioner, *ED* Emergency Department

^a^3 responses were also classified as “consultation for an administrative reason”

^b^1 response was also classified as “consultation for drug-seeker” and 1 response was also classified as “consultation for administrative reason”

For the client satisfaction survey, the majority of clients (59%; 129) rated their visit as 10 (very satisfied), and a combined total of 190 (86%) rated their visit as 8, 9 or 10 (Table 3). Seventy-nine per cent (79%; 174) said their health issue was resolved as an outcome of their visit. Of the 45 (21%) issues that were not resolved, within one week of presenting the BAHGPC, 73% (33) saw another GP, 22% (10) went to ED and 9% (4) did not seek additional medical assistance. Ninety-nine per cent (99%; 216) of respondents said that would use the clinic again and 99% (217) would recommend the clinic to someone else (Table 3).

**Table 3:** Client satisfaction follow-up survey

	Number	Percentage (%)
Client satisfaction with the visit (*n* = 219)		
10 (very satisfied)	129	59%
9	40	18%
8	21	10%
7	15	7%
6	4	2%
5	8	4%
4	1	0%
3	0	0%
2	0	0%
1	1	0%
0 (very dissatisfied)		
Was the clients issue resolved: (n = 219)		
Yes	174	79%
No	45	21%
Saw another GP within one week of visiting the After Hours clinic (n = 45^a^)^b^	33	73%
Went to ED within one week of visiting the After Hours clinic (*n* = 45)^a^	10	22%
Did not anyone else within one week of visiting the After Hours clinic (n = 45)	4	9%
Would the client use the clinic again: (n = 219)		
Yes	216	99%
No	3	1%
Would the client recommend the clinic to someone else: (n = 219)		
Yes	217	99%
No	2	1%

*Abbreviations*: *GP* General Practitioner, *ED* Emergency Department

^a^2 respondents saw another GP *and* went to ED within one week of visiting the After Hours clinic

^b^The after hours GP routinely encourages clients to make an appointment with their regular GP for the week following the BAHGPC consultation

Of the 219 participants that completed the follow-up survey, 162 (74%) provided additional comments about the service when asked. Almost all respondents gave positive comments about the BAHGPC (90%; 147), such as: *“Service is very good and it's wonderful to have in town. Also relieves the Emergency Department. Think it's absolutely marvellous”*, and *“Absolutely excellent service! Nothing worse than having to go to Emergency when “you’re not dying”. Well-needed service in Bathurst”.* Only 5 (3%) respondents gave negative comments about the service which were due to: a lack of equipment, a lack of privacy at reception with personal details being relayed to the client, a long waiting time, and being turned away from the service at 6.45 pm (when the clinic closes at 7 pm). An additional two respondents provided a negative comment about the general practitioner on call, but also said that the BAHGPC was a good service. There were 13 (8%) comments that were neither positive nor negative, for example, *“Would not use the clinic again because have moved to Sydney”.*

Some respondents gave recommendations for improvement which included: the clinic being open for longer hours (12), more advertising about the clinic (2), the clinic being connected to an after hours pharmacy (2), having an extra GP at the clinic to decrease waiting times (1), and more infrastructure and equipment (1).

### Presentations to the bathurst base hospital emergency department

Non-urgent and semi-urgent presentations to the Bathurst ED in the two years prior to the BAHGPC opening and in the subsequent two years were compared both in terms of raw numbers and on a population basis (Table 4). There was a significant reduction in non-urgent ED presentations in the two years following the opening of the BAHGPC as compared to the two years prior (418.5 vs 245.5; *P* < 0.05). There was no significant reduction in semi-urgent ED presentations in the same period. On a population basis, in the two years prior to the opening of the BAHGPC non-urgent presentations equated to 1.0% of the Bathurst population (Table 4). This dropped significantly in the two years following the opening of the clinic to 0.6% (*P* < 0.05). There was no significant difference in semi-urgent ED presentations in this period on a population basis.

**Table 4:** Emergency Department and After Hours Clinic presentations pre and post commencement of the Clinic

	2 years prior to after hours clinic	2 years subsequent to after hours clinic
Non-urgent and semi-urgent presentations		
Total numbers of people		
Non-urgent presentations (Mean ± SD)	418.5 ± 9.19	245.5 ± 27.58*
Semi-urgent presentations (Mean ± SD)	3984.5 ± 47.38	3931.5 ± 81.32
As a percentage of the Bathurst Population**		
Non-urgent presentations (Mean ± SD)	1.0 ± 0.03	0.6 ± 0.07*
Semi-urgent presentations (Mean ± SD)	9.9 ± 0.05	9.5 ± 0.30
Presentations by gender		
Number (percentage) of non-urgent presentations by gender		
Females	359 (42.9%)	208 (42.4%)
Males	478 (57.1%)	283 (57.6%)
Number (percentage) of after hours clinic presentations by gender***		
Females		1205 (56.7%)
Males		920 (43.2%)

*Indicates value is significantly different (*P <* 0.05) to the corresponding value for the two years prior to the clinic opening

**Source: Australian Bureau of Statistics [19]

***Proportion of females to males significantly different (X^2^ 41.45; *P <* 0.05) to their respective proportions in non-urgent presentations in the two years following the opening of the clinic

Non-urgent ED presentations in the two years prior to the opening of the after hours clinic and the two years subsequent to the opening of the clinic were stratified by gender and compared to the number of visits to the after hours clinic in the first two years of operation (Table 4). More males than females presented to the ED with non-urgent complaints during the first two years of the BAHGPC being in operation. In contrast, more females than males attended the BAHGPC in this same period. This difference in proportions was significantly different (*P* < 0.05).

## Discussion

After hours GP clinics are intended to relieve pressure on EDs by diverting suitable clients to a primary care alternative. They are designed to cater for people who do not have an emergency situation but who are seeking immediate treatment due to the perceived urgency of their complaint, and are hence unable to wait to see their regular general practitioner during routine opening hours. The primary aim of this study was to examine the appropriateness of presentations to an after hours GP clinic from both the client and the general practitioner perspective and to examine client satisfaction with the clinic. A secondary aim was to examine changes in non-urgent and semi-urgent ED presentations in the two years before, and following, the opening of the after hours clinic.

Clients and doctors can have different perceptions of the level of urgency of a medical complaint, leading to different interpretations of the appropriateness of a presentation to an ED and/or after hours clinic [13, 14]. In this study, we found good concordance between clients and doctors as to the necessity and appropriateness of presentations to the BAHGPC. Seventy-six per cent (76%) of clients deemed their visit as essential whilst general practitioners ranked 87% of presentations as appropriate and 86% as being in the range of moderately to absolutely essential. To our knowledge, only one other study has compared the perceptions of clients and doctors regarding the appropriateness of presentations to an after hours service [12]. They found that nearly two-thirds of non-urgent contacts to an after hours GP cooperative were “medically unnecessary”, despite the clients themselves believing their presentation to be urgent, and could have been readily managed by the client or by their regular general practitioner during routine working hours [12]. The authors suggested that the purpose and intention of the GP cooperative needs to be made clearer to the general public so that they can reduce the number of medically unnecessary contacts that are occurring. In our study, the low percentage of presentations identified by general practitioners on the “not appropriate” end of the scale (14%) suggests that the BAHGPC is being utilised for the purpose of servicing low acuity clients who might otherwise have presented to the ED. As this relatively new service becomes more established in this location it will be important to monitor future trends to ensure the rate of inappropriate presentations to the clinic remains low.

In this regional location, where after hours options are currently limited solely to the BAHGPC, 60% of respondents indicated they would have presented to the local ED had the BAHGPC not been operating on that day. Based on this data alone, we can surmise that the BAHGPC is having a positive impact on reducing unnecessary ED presentations. Certainly, data on ED presentations showed a significant reduction in non-urgent, but not semi-urgent, ED presentations in the two years since the opening of the BAHGPC as compared to the two years prior to this. This trend was maintained even when accounting for population changes that occurred in those time periods. International evidence regarding the impact of after hours clinics on ED presentations is equivocal and differences in health system structures and after hours service delivery make direct comparisons difficult. Australian evidence is limited, but is also equivocal. One urban study of an after hours clinic found a potentially positive impact on unnecessary ED attendances, however they relied solely on client reports of where they would have sought treatment had the after hours clinic not been an option, rather than examining ED presentations directly [15]. In contrast, another urban based study modelled the impact of a hypothetical after hours clinic on ED presentations and concluded that the clinic would be unlikely to produce any remarkable impact on ED presentations [16]. More recently, in a regional location similar to this study, researchers used regression time series analysis to assess the impact of opening an after-hours GP clinic and found a significant reduction in low-urgency ED presentations with the opening of the clinic [17]. Similarly to our study, the above study was conducted in a regional location where after hours options are limited to the clinic and the ED. Unlike the clinic in our study however, their after hours clinic was not a bulk-billing clinic and clients pay to use their service. Notably, in our study three-quarters of respondents indicated a willingness to pay to use the BAHGPC and those with private health insurance were more likely to indicate a willingness to pay.

A significant difference was detected in the proportion of females and males presenting to the BAHGPC versus those presenting to the ED with non-urgent complaints. Females were the majority in after hours clinic presentations whilst males made up the majority in non-urgent ED presentations. This finding concurs with other studies which have also shown that females were more likely to use after hours options than males who tended towards the ED [12, 13, 18]. We did not record gender on either our client presentation survey or the general practitioner appropriateness of presentation survey, so cannot determine from the data if a difference exists between males and females regarding the type of health issue they were presenting to the BAHGPC with. Similarly, no such analysis was possible on the ED data that we retrieved. This represents an avenue for future research to determine why males are more likely to present to the ED and examine if their health issues are suitable for treatment at the BAHGPC.

### Strengths and limitations

Strengths of this study include that the data regarding client and general practitioner perceptions of the appropriateness and necessity of the presentation were completed at the time of presentation. In the case of the general practitioner assessment of the appropriateness of the presentation, this enabled them to draw on their interaction with the client as part of this process rather than making a retrospective assessment of suitability based on case notes or a client survey, as has been the case in previous research. Another strength is that the clinic examined in this study is a bulk-billing, walk in clinic so there are no apparent access issues for people relating to their socioeconomic status and ability to pay for the service. In addition, it is the only after hours option available in this community, thereby strengthening the conclusions that can be drawn from an examination of ED presentations since the opening of the clinic. It should be acknowledged that there are other factors that can influence ED presentation rate beyond the presence of the BAHGPC alone. However, the absence of other after hours options in this community coupled with the data from this study suggests that the presence of the BAHGPC is having some positive impact on reducing non-urgent ED presentations. Another strength of this study is that we collected both client and general practitioner data regarding the necessity of the presentation. A limitation of this data though is that there was no linkage between the client data and the general practitioner data, therefore we are not able to determine if the participating clients are indeed represented in the general practitioner data. Furthermore, given that clients volunteered to participate and we only obtained a 25% response rate to the client presentation survey, it is possible that the results are not representative of all clients accessing the BAHGPC. Another potential limitation is that the ED presentation data was obtained from 12:00 pm Saturday through to 8:00 am Monday as this is the time-frame over which there are no GP clinics open in Bathurst. However, the BAHGPC is only open for some of these hours (3:00 pm – 7:00 pm Saturday and Sunday). Whilst it is likely that some clients were happy to wait until the BAHGPC was open rather than attend the ED, it is equally as likely that some prospective BAHGPC clients chose to attend the ED rather than wait for the BAHGPC to open. Further research could explore this in greater detail.

### Implications

The current research adds to an equivocal evidence base regarding the effectiveness of after hours primary care clinics both nationally and internationally. The positive impact of the after hours clinic seen in this study is likely facilitated by the presence of this bulk-billing, walk in clinic in a regional centre where no other after hours options exist beyond the local ED. This evidence may inform the targeting and implementation of other after hours clinics in similar regional or remote locations to optimise healthcare expenditure on low acuity clients. It is important to note, however, that our findings may not be generalisable to all regions of Australia, particularly metropolitan regions. The current published differences regarding the impact of after hours GP clinics on local ED presentations in Australia may reflect true differences between regions with respect to demand and accessibility. A critical factor at play here could be workforce. Rural and regional areas of Australia, such as Bathurst, are typically identified as areas of general practitioner workforce shortage, as compared to most metropolitan settings which do not bear this label. Hence, the impact of an after hours GP clinic in a rural/regional setting may be more significant in terms of influencing local ED presentations than it would be in metropolitan centres with ample general practitioners and the presence of numerous GP clinics with the capacity to offer after hours services to their clients. Future research could focus on these issues in more depth.

In addition, future research where direct linkage is performed between client perceptions and general practitioner perceptions regarding necessity and appropriateness of a presentation to after hours clinics would add to the evidence base on this topic. Such data can inform client education initiatives to ensure that after hours clinics continue to be used for the purpose for which they were set up – to divert low acuity clients away from the ED – without being burdened by medically unnecessary consultations, borne out of convenience or a clients misguided sense of urgency, that need not have occurred immediately in the after hours period.

## Conclusions

The BAHGPC is providing a much needed and appropriately-utilised after hours primary care service to the Bathurst community, that is contributing to reducing the burden of non-urgent presentations to the local ED in this regional centre. The findings highlight the necessity of such a service to rural and regional communities, and regions where there is an identified shortage in the general practitioner workforce, where the impact of demand and accessibility issues is most profound.

## Additional files

Additional file 1: Client Presentation Survey (PDF 170 kb)

Additional file 2: Client Follow-up Survey (PDF 78 kb)

Additional file 3: GP Appropriateness of Presentation Survey (PDF 133 kb)

## Abbreviations

BAHGPC: Bathurst After Hours General Practice Clinic
ED: Emergency department
GP: General practice
