# Supplementary material for: An after hours gp clinic in regional Australia: appropriateness of presentations and impact on local emergency department presentations
Source: BMC Fam Pract. 2017 Sep 11;18:86. doi: 10.1186/s12875-017-0657-6 (PMC5594615; doi:10.1186/s12875-017-0657-6)
Supplement: Supplementary file 1 — Client Presentation Survey (PDF 170 kb) [file 12875_2017_657_MOESM1_ESM.pdf]

Patient survey

medicare local
WESTERN NSW
Connecting health to meet local needs

The after hours service is looking to capture the patient's opinion on the four (4) questions below. To assist us with this, we are asking Clinic Nurses to ask the patients these questions and notate the answers on this form for each patient – *thank you.*

Question 1.

What is the patient's reason for attending the GP After Hours Clinic?
(Most appropriate main reason)

a) Essential visit (had to come)
b) Only to get a script (out of my medication)
c) Couldn't get an appointment with a regular GP
d) Requested by the GP to attend
e) Requested by the hospital ED to attend
f) Convenient
g) Other --

Question 2.

If the after hours clinic was not functioning today, the patient would

a) Go to the Hospital ED
a) Try and consult another GP in town
b) Wait until next week to see the patients own GP
c) Visit a pharmacist and try to get medication
d) Other--

Question 3.

If the patient had to pay for the service they receive at the after hours clinic, would they use the service?
(circle one only)

a) Yes
b) No

If yes, what is the maximum you would be willing to pay?
(circle one only)

a) A small co-payment (less than $10)
b) The same amount you would pay at your GP
c) More than what you would pay at your GP

If no, please provide a reason in the space below (optional) ---

----

Question 4.

Does the patient have:

a) a Health Care Card YES NO b) Private Health Insurance YES NO

Please thank the patient for their participation. V2. 14th February
