# Supplementary material for: An after hours gp clinic in regional Australia: appropriateness of presentations and impact on local emergency department presentations
Source: BMC Fam Pract. 2017 Sep 11;18:86. doi: 10.1186/s12875-017-0657-6 (PMC5594615; doi:10.1186/s12875-017-0657-6)
Supplement: Supplementary file 2 — Client Follow-up Survey (PDF 78 kb) [file 12875_2017_657_MOESM2_ESM.pdf]

Patient - Post Clinic follow-up questionnaire

medicare local
WESTERN NSW
Connecting health to meet local needs

As a way to ensure that the After Hours Service continues to meet the needs of the community, we are looking to capture the views of patients who have recently attended the After Hours GP Clinic.

Without telling me any personal information about your visit to the GP Clinic, could you please answer the following four (4) questions?

Question 1.

a) Would you say In general that your health is?
(please circle only one)

i) Excellent   ii) Very good   iii) Good   iv) Fair   v) Poor

Question 2.

a) On a scale from 1 to 10, with 10 being very satisfied and 1 being very dissatisfied, how would you rate your satisfaction with the outcome of your visit to the GP After Hours Clinic?
(*please circle)*

1   2   3   4   5   6   7   8   9   10

Question 3.

a) Was the reason you attended the GP Clinic resolved at the time of visit?

a) YES   b) NO

b) if no, within 1 week of attending the clinic for the initial issue, did you:

i) see another GP   ii) go to the ED   iii) see someone else (e.g. pharmacy) ---------------------

vi) didn't see anyone else

Question 4.

a) Would you use this service again? b) Would you recommend this service to others

a) use self   YES   NO   **b) recommend**   Yes   No

Is there anything that you would like to add or make comment on?

Please thank the patient for their participation. **V1 16th February 2015**
