# Supplementary material for: An after hours gp clinic in regional Australia: appropriateness of presentations and impact on local emergency department presentations
Source: BMC Fam Pract. 2017 Sep 11;18:86. doi: 10.1186/s12875-017-0657-6 (PMC5594615; doi:10.1186/s12875-017-0657-6)
Supplement: Supplementary file 3 — GP Appropriateness of Presentation Survey (PDF 133 kb) [file 12875_2017_657_MOESM3_ESM.pdf]

medicare local
WESTERN NSW
Connecting health to meet local needs

GP Survey on the appropriateness of presentations to the after hours clinic

The after hours service is looking to capture the views of the Clinic Doctor on the appropriateness of presentations to the after hours clinic. To assist us with this, it would be appreciated if you could answer the two (2) questions below for each patient.

Question 1.

What is the reason for this presentation?

Primary reason for encounter: --

Primary problem definition:---

Question 2.

a) Please indicate your opinion of the appropriateness of this patient's presentation by circling the corresponding number below
(*please circle one)*

(Not appropriate) **0** **1** **2** **3** **4** **5** (Absolutely essential)

b) Visit classification
(Please circle one or more)

a) Consultation was appropriate

b) Consultation was appropriate and needed a referral to hospital/ED

c) Consultation was appropriate as it was requested by the patients GP during the week

d) Consultation only for a script

e) Consultation for an administrative reason – e.g. medical certificate

f) Consultation for drug-seeker

g) Consultation because it was convenient for the patient

h) Other:---

Thank you for your participation. **v1 December 2014**
